# Effects of cold-water immersion at different body regions on post-exercise muscle damage recovery: a systematic review and meta-analysis

**DOI:** 10.3389/fspor.2026.1738075

**Published:** 2026-02-05

**Authors:** Yang Zhu, Lele Yang, Tao Liu, Fuya Yao, Qilong Wang, Zheng Yi

**Affiliations:** College of Physical Education and Training, Capital University of Physical Education and Sports, Beijing, China

**Keywords:** cold therapy, delayed onset muscle soreness, explosive power, muscle damage, muscle strength, physical training, post-exercise recovery

## Abstract

**Objective:**

The aim of this study was to systematically evaluate the differences in efficacy of Cold-Water Immersion (CWI) applied to different body regions for post-exercise muscle damage recovery.

**Methods:**

The PRISMA guidelines were followed. Databases including PubMed, Embase, Web of Science, and the Cochrane Library were systematically searched from inception to October 20, 2025. Randomized controlled trials (RCTs) comparing single acute CWI with seated passive rest were included. Methodological quality and risk of bias were assessed using the PEDro scale and RoB 2 tool, respectively. Data analysis was performed using Stata-MP 18.0 software.

**Results:**

Thirty RCTs were included, and the overall quality was high. The meta-analysis showed that CWI significantly reduced post-exercise CK levels (g = –0.24; 95% CI: −0.37 to −0.10, *P* < 0.01) and alleviated DOMS (g = –0.40; 95% CI: −0.64 to −0.16, *P* < 0.01) compared to the control group. However, no statistically significant benefits were observed for the recovery of CMJ (g = –0.02; 95% CI: −0.17 to 0.13, *P* > 0.05) or MVIC (g = 0.08; 95% CI: −0.08 to 0.23, *P* > 0.05). Subgroup analysis indicated no significant difference between whole-body and partial immersion at any follow-up time point (0–72 h) (between-group *P* > 0.05). Notably, a significant inhibitory effect on immediate (0 h) explosive power (CMJ) was shown by partial CWI (g = –0.94, *P* < 0.01). The primary biochemical and subjective benefits were concentrated at 24 h. Robustness of the main results was confirmed by sensitivity analysis, though potential publication bias was detected for CK.

**Conclusion:**

Biochemical (CK) and subjective (DOMS) recovery were effectively improved by CWI. However, muscle strength (MVIC) was not synchronously enhanced, and explosive power (CMJ) was immediately inhibited. The equivalence of therapeutic efficacy between whole-body and partial immersion was confirmed as a core finding. Consequently, partial immersion is considered the optimal strategy combining efficacy and safety. It is suitable for accelerating next-day recovery. However, it should be used with caution during intervals involving continuous explosive power output.

**Systematic Review Registration:**

PROSPERO CRD420251171826.

## 1 Introduction

Skeletal muscle is considered the fundamental tissue of the human movement system (1). Exercise-induced muscle damage (EIMD) is often induced by high-intensity or unaccustomed exercise (especially modes involving extensive eccentric contractions). This process is regarded as a common and recurrent physiological phenomenon in competitive sports (2). Its pathological basis involves mechanical disruption of muscle fiber ultrastructure (such as sarcomere disorganization). Intracellular calcium homeostasis is disturbed. A secondary inflammatory cascade is subsequently triggered. Clinically and functionally, delayed onset muscle soreness (DOMS) and elevated circulating sarcoplasmic enzymes (such as creatine kinase, CK) are typically observed. Temporary inhibition of maximal strength and explosive power is also manifested (3, 4). During intensive training cycles or congested match schedules, fatigue accumulation is exacerbated if EIMD recovery is insufficient. Subsequent athletic performance may be significantly impaired. The risk of musculoskeletal injury is increased (5, 6). Therefore, the quantification of the recovery trajectory through objective indicators is considered crucial. Among various recovery indicators, muscle strength recovery is given a core position (7). It serves as the basis for athletic performance and motor control. It is also regarded as a key reference for training load management (8). A multidimensional assessment system is often established in research and practice. Basal muscle strength is assessed by maximal voluntary isometric contraction (MVIC). Neuromuscular explosive power is reflected by the countermovement jump (CMJ) (9). Serum CK levels are primarily used as biochemical markers of tissue damage (10). Subjective pain sensation (i.e., DOMS) is assessed by the Visual Analog Scale (VAS) (11). These complementary indicators are widely used to evaluate the clinical value and immediate effects of various recovery interventions.

To accelerate the recovery process, cold-water immersion (CWI) has become one of the most commonly applied interventions in sports science. Its therapeutic mechanisms include the reduction of tissue temperature and the induction of vasoconstriction. Inflammatory responses are alleviated. Neuromuscular activation patterns are altered. Consequently, exercise-induced muscle damage is mitigated and recovery is accelerated (12). Although CWI is widely recommended for alleviating DOMS and reducing CK levels, its efficacy for recovering muscle strength and explosive power remains controversial. High heterogeneity was observed among different studies. Early systematic reviews, such as Machado et al. (13), focused primarily on “dosage” parameters. An intervention standard of 11–15 °C for 11–15 min was preliminarily established. However, while the overall effectiveness of CWI was confirmed by the systematic review of Malta et al. (14), significant variations in explosive power recovery were noted across studies. This inconsistency was also reflected in the study by Moore et al. (5). Although perceived recovery was effectively improved by CWI, positive effects on muscle strength recovery following eccentric exercise were limited to within 24 h. Furthermore, a potential negative interference with performance in the immediate term (time-dependency) was suggested by Garcia et al. (15). Long-term cold therapy was found to inhibit satellite cell activity and the mTOR signaling pathway by Roberts et al. (16), thereby impairing muscle hypertrophy adaptation. Methodological support for optimal dosage selection was provided by the recent network meta-analysis of Wang et al. (17). However, unexplained differences in effect sizes were still noted even under similar temperature dosages. This suggested that other key effect modifiers might have been overlooked.

A critical factor for these discrepancies was identified as the focus of existing reviews on temperature and duration. The anatomical distribution of cold stimulation (i.e., immersion region/depth) was largely ignored as a key effect modifier. Therefore, CWI was categorized into two types based on anatomical landmarks in this study: whole-body immersion (water level above the iliac crest, including navel, sternum, or neck level) and lower-body immersion (water level at or below the iliac crest) (18). This classification was based on differences in three core physiological mechanisms. First, the hydrostatic pressure effect: specific hemodynamic changes are induced. A higher hydrostatic pressure gradient is generated by whole-body immersion. Central blood volume is increased and venous return is promoted. In contrast, weaker hydrodynamic effects are observed in lower-body immersion (19, 20). Second, the thermal conduction rate: a larger surface area for heat exchange is involved in systemic immersion. Core body temperature is reduced more rapidly (21). Third, autonomic regulation: the vagus nerve is more strongly activated by deep water immersion involving the neck/chest. Specific regulation of heart rate variability and subjective recovery is induced (22).

Based on this evident gap in evidence, a systematic review and meta-analysis was conducted. The specific aim was to explore the effects of CWI at different body regions (whole-body vs. partial) on post-exercise muscle damage recovery across specific follow-up time points (0 h, 24 h, 48 h, and 72 h). Four key indicators (MVIC, CMJ, CK, and DOMS) were synthesized. The differences in recovery efficacy between different immersion depths were quantitatively compared. An attempt was made to address the systematic gap regarding the “anatomical dimension” in CWI research. Evidence-based references for the formulation of more precise and anatomically grounded cold therapy protocols in sports rehabilitation are provided.

## 2 Materials and methods

### 2.1 Registration

The study protocol was registered on PROSPERO (Registration number: CRD420251171826). The systematic review and meta-analysis followed the PRISMA guidelines strictly (23). This ensured transparency and consistency. According to the classification by Ato et al. (2013), this study is a theoretical study (24). The methodology leader (ZY) independently designed the process and monitored quality to ensure scientific rigor.

### 2.2 Literature search strategy

**Figure 1:**
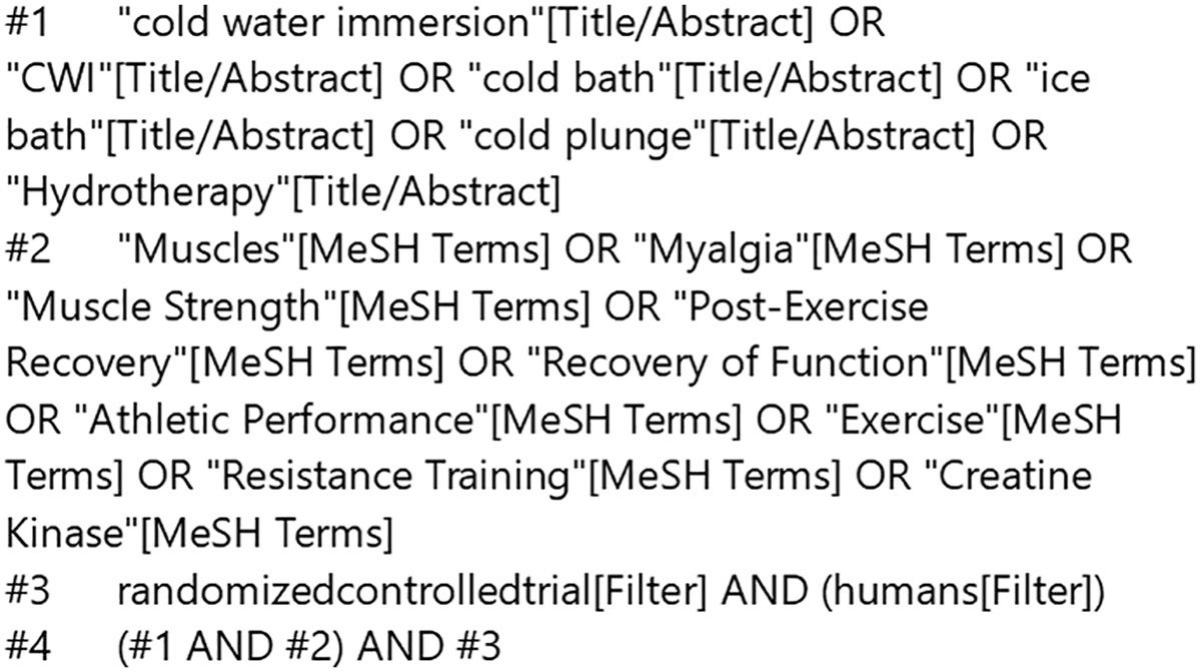
PubMed search formula.

Computer searches were conducted in databases including PubMed, Web of Science, Cochrane Library, and Embase. The literature search period spanned from the inception of each database to October 20, 2025. In addition to the electronic database searches, we manually screened the reference lists of all included studies and relevant systematic reviews to identify further eligible studies. A strategy combining subject headings with free-text terms was employed for literature retrieval. Using PubMed as an example, the search strategy is illustrated in Figure 1, while detailed search strategies for the remaining databases are provided in the Supplementary Materials.

### 2.3 Inclusion and exclusion criteria

The inclusion and exclusion criteria for literature were established according to the Participants, Interventions, Comparisons, Outcomes, and Study Design (PICOS) framework (25).

#### 2.3.1 Inclusion criteria

Conducted in healthy individuals with no recent illness or history of chronic disease.
CWI intervention performed within 1-hour post-exercise with reported CWI parameters.
Control group engaged in seated rest.
Outcomes reporting at least one of CK, DOMS, CMJ, or MVIC.
Randomized controlled trials.

#### 2.3.2 Exclusion criteria

Multiple CWI interventions or ambiguous CWI parameters.
Only studies using cold water immersion (CWI) as the sole intervention; studies combining CWI with other recovery methods (e.g., active recovery, stretching, massage) or nutritional supplements are excluded (26).
Studies where valid outcome data could not be extracted and authors could not be reached for clarification.
Case reports, cohort studies, qualitative studies, systematic reviews or meta-analyses, study protocols, preprints not peer-reviewed for publication, and conference abstracts.
Duplicate publications.

### 2.4 Literature screening

Search results were managed using EndNote X9 software, with duplicates removed by an independent reviewer (YZ). Subsequently, two reviewers (LL, TL) independently screened titles and abstracts against the inclusion criteria. Studies failing to meet eligibility standards were excluded, and full-text articles were retrieved for detailed evaluation. Any discrepancies were resolved through discussion to reach consensus, with a third reviewer (ZY) adjudicating if necessary. Two reviewers independently extracted and cross-checked the data; inconsistencies were verified by a third reviewer. In instances of missing or incomplete data, corresponding authors were contacted to ensure data integrity.

### 2.5 Data extraction

Literature data extraction was performed independently by two researchers using a double-blind method. A standardized template created in Microsoft Excel was employed to extract the following information: 1. Basic details: First author, publication date, study type, study population (sample size, gender, age, height, weight); 2. Study design: Exercise type and protocol, CWI protocol, outcome measures, intervention duration.

### 2.6 Bias risk and quality assessment

The risk of bias was assessed using the Cochrane Risk of Bias Tool 2 (RoB 2) (27). The assessment encompassed five key domains: randomization process, deviations from intended interventions, missing outcome data, measurement of the outcome, and selection of the reported result. Disagreements were resolved through discussion; failing consensus, a third reviewer served as an arbitrator. Risk of bias visualization plots were generated using the robvis Shiny web app (28).

To supplement the quality assessment, the Physiotherapy Evidence Database (PEDro) scale was utilized (29). The PEDro scale has a total of 10 points. The criteria were: ≥6 points (high quality), 4–5 points (moderate quality), and ≤3 points (low quality). PEDro scores were employed to quantify bias risk and study design quality, thereby facilitating the interpretation of the robustness of the pooled results.

### 2.7 Statistical analysis

All statistical analyses were conducted using Stata-MP 18. To minimize confounding from baseline differences, change scores (i.e., the mean differences before and after the intervention along with their standard deviations) were used as the effect size input. Standardized mean differences [SMD (30)] were calculated, and Hedges' g was applied to correct for small-sample bias. All pooled effects are reported as Hedges' g with corresponding 95% confidence intervals. Effect sizes were interpreted according to Cohen's classification (31): large effect (g > 0.8), moderate effect (0.5 ≤ g ≤ 0.8), small effect (0.2 ≤ g ≤ 0.5), and trivial effect (g ≤ 0.2). Descriptive statistics for continuous variables are presented as mean ± standard deviation (mean ± SD). Statistical significance was set at a two-sided *P* < 0.05.

Heterogeneity was assessed using Cochran's *Q* test and the I^2^ statistic. Significant heterogeneity was defined as *p* < 0.10 or I^2^ > 50%, in which case a random-effects model was employed; otherwise, a fixed-effect model was applied. To explore potential sources of heterogeneity, stratified subgroup analyses were conducted based on immersion regions at specific follow-up time points (0 h, 24 h, 48 h, and 72 h). Sensitivity analyses were conducted using a leave-one-out approach to evaluate the robustness of pooled effects (32). Publication bias was assessed both qualitatively, via funnel plot inspection, and quantitatively, using Egger's regression test. Potential publication bias was considered present if Egger's test *P* < 0.05 or if funnel plot asymmetry was evident. To further validate the reliability of the results, the trim-and-fill method proposed by Duval and Tweedie was applied to adjust for possible publication bias and recalculate the effect sizes (33).

## 3 Results

### 3.1 Literature search results

**Figure 2:**
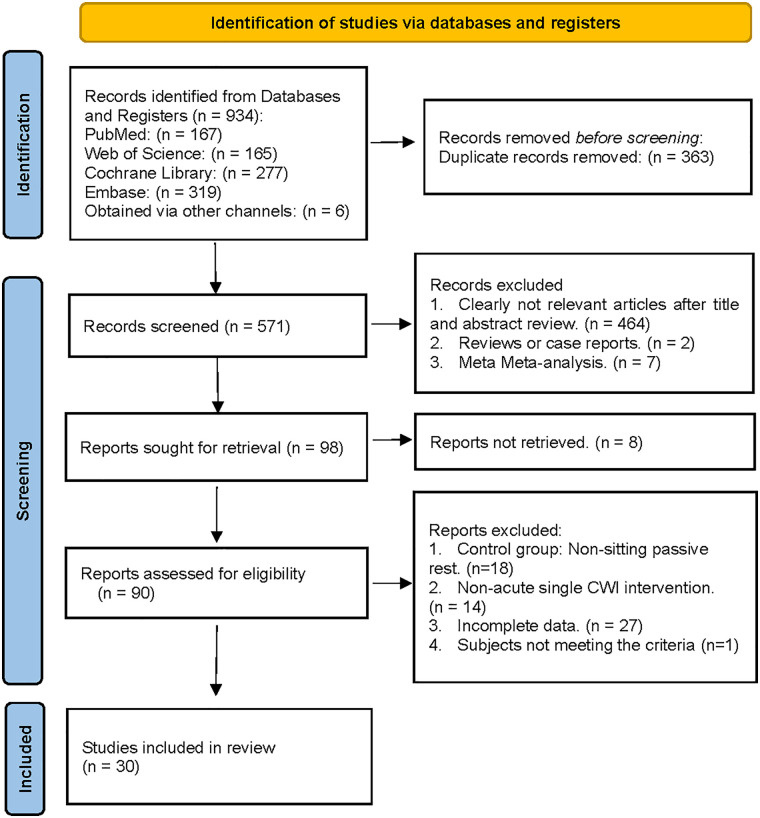
Flow diagram of the selection process.

Based on the predefined search strategy, a total of 928 articles were retrieved from multiple databases. Through manual reference verification, an additional 6 articles were included, bringing the total to 934. After deduplication using EndNote X9 software, 363 articles were excluded, leaving 571. Following screening of titles and abstracts, 473 articles were excluded, resulting in 98 articles advancing to full-text review. During full-text review, 60 studies were excluded. An additional 8 studies were excluded due to unavailability of full-text. Ultimately, 30 studies met the inclusion criteria and were used for quantitative meta-analysis (Figure 2).

### 3.2 Basic characteristics and information of included literature

This study includes a total of 30 research articles, published between 2008 and 2025. The total sample size across all studies is 527 participants (498 male, 32 female), with sample sizes ranging from a minimum of 7 participants (34) to a maximum of 60 participants (35). The age range of participants spans from 15.6 to 50.3 years. All studies included are randomized controlled trials, with 15 studies employing a parallel-group design (3, 35–48), and 15 studies utilizing a crossover design (4, 34, 49–61). In the crossover studies, a washout period was implemented to minimize carryover effects, ensuring that participants' physiological states, muscle damage markers, and subjective symptoms returned to baseline or stabilized before entering the next intervention phase. This approach ensured the comparability of results across intervention periods (62).

Among the included studies, 12 focused on healthy, non-athlete participants (3, 4, 35–37, 42, 43, 47–49, 56, 60), while the remaining 18 involved professional athletes (34, 38–41, 44–46, 50–55, 57–59, 61). The distribution of studies by type of exercise is as follows: 12 studies investigated eccentric or eccentric-concentric resistance training (3, 34–37, 39, 41, 43, 48, 52, 56, 59); 15 studies focused on high-intensity interval training (HIIT), including sprint running, LIST/Yo-Yo tests, and simulated competition/training (4, 38, 44–46, 49–51, 53–55, 57, 58, 60, 61); and 3 studies examined aerobic endurance exercises (40, 42, 47). All studies reported adverse events, with specific details provided in Tables 1, 2.

**Table 1:** Basic information of the literature included.

Included studies	Country	Research type	Research subjects	Age (years)	Sample size		Height/Weight (kg/cm)
					CWI	CON	
Sánchez 2018 (36)	CR	PS	Healthy male	21.8 ± 2.8	13M	13M	176.6 ± 5.3/73.2 ± 8.2
Amir 2017 (37)	MY	PS	Healthy male	21.6 ± 2.3	8M	8M	167.2 ± 6.4/61.6 ± 11.1
Anderson 2017 (38)	UK	PS	Team event athletes	24.0 ± 2.0	9M	9M	178.0 ± 9.0/77.6 ± 14.2
Angelopoulos 2022 (39)	GER	PS	Athlete	21.1	15M	15M	176.0/77.6
Broatch 2014 (49)	AU	CS	Healthy male	24.0 ± 5.0	10M	10M	179.3 ± 6.6/78.7 ± 8.5
Crowther 2017 (4)	AU	CS	Healthy male	27.0 ± 6.0	29M	29M	180.0 ± 8.0/80 ± 9
Dantas 2019 (40)	BR	PS	Male runners	31.6 ± 3.9	10M	10M	175.6 ± 6.3/77.7 ± 7.0
Doeringer 2017 (41)	UK	PS	Healthy athletes	20.7 ± 1.40	4M/8F	3M/7F	170.9 ± 5.2/68.2 ± 8.4
Elias 2012 (50)	AU	CS	Soccer players	20.9 ± 3.3	14M	14M	186.0 ± 7.2/79.6 ± 6.7
Fonseca 2016 (51)	BR	CS	Jiu-jitsu athlete	24.0 ± 3.6	8M	8M	NR/78.4 ± 2.4
Haq 2022 (42)	UK	PS	Healthy male	37.0 ± 13.3	8M	8M	176.0 ± 7.0/79.5 ± 13.7
Heinke 2024 (43)	GER	PS	Healthy participants	24.3 ± 3.5	8M/3F	9M/3F	178.0 ± 9.0/75.6 ± 12.6
Higgins 2013 (44)	AU	PS	Rugby player	19.4 ± 0.8	8M	8M	178.5 ± 5.7/82.4 ± 11.1
Kim 2023 (52)	KR	CS	Soccer players	25.4 ± 5.2	11M	11M	182.3 ± 2.0/76.3 ± 1.5
Leeder 2015 (45)	UK	PS	Athlete	23.0 ± 3.0	8M/8M	8M	NR/81.4 ± 8.7
Li 2023 (53)	CN	CS	Basketball player	22.80 ± 0.84	10M	10M	179.0 ± 4.0/75.6 ± 6.6
Machado 2016 (35)	BR	PS	Healthy male	20.8 ± 2.15	20M/20M	20M	174.0 ± 5.0/74.4 ± 11.2
Minett 2013 (54)	AU	CS	Team sport athletes	21.0 ± 2.0	9M	9M	183.3 ± 7.0/78.7 ± 8.1
Nasser 2023 (55)	TN	CS	Soccer players	21.1 ± 2.2	12M	12M	174.9 ± 4.6/72.4 ± 5.9
Ottone 2022 (56)	BR	CS	Healthy male	24.0 ± 5.0	11M	11M	178.0 ± 7.0/73.0 ± 13.3
Pesenti 2020 (34)	BR	CS	Soccer players	16.5 ± 0.9	7M	7M	174 ± 5.2/69.2 ± 5.1
Pointon 2012 (57)	AU	CS	Male rugby players	21.0 ± 1.7	10M	10M	182.9 ± 6.1/87.2 ± 7.7
Rupp 2012 (46)	UK	PS	Soccer players (13M/9F)	19.8 ± 1.1	12	10	174.0 ± 9.0/72.1 ± 9.1
Silva 2017 (58)	BR	CS	Jiu-jitsu athlete	21.7 ± 3.1	10M2F	10M2F	170.0 ± 5.0/72.1 ± 13.0
Takeda 2014 (61)	JP	CS	Male rugby players	20.3 ± 0.6	20M	20M	174.0 ± 5.0/85.4 ± 2.0
Valle 2008 (59)	AU	CS	Male strength trainer	NR	12M	12M	NR
Wei 2025 (3)	CN	PS	Healthy male	21.1 ± 1.4	5M	5M	175.7 ± 4.5/69.5 ± 6.8
White 2014 (60)	CA	CS	Healthy male	23.6 ± 3.7	8M	8M	180.8 ± 8.1/76.1 ± 8.6
Wiewelhove 2018 (47)	GER	PS	Male long-distance runner	30.5 ± 10.9	11M	12M	179.4 ± 6.2/75.5 ± 7.5
Yoshimura 2023 (48)	JP	PS	Healthy male	21.4 ± 0.8	8M	10M	171.7 ± 7.9/64.5 ± 7.6

CWI, cold water immersion group; CON, resting in seated meditation group; CR, Costa Rica; MY, Malaysia; UK, United Kingdom; GER, Germany; AU, Australia; BR, Brazil; KR, South Korea; CN, China; TN, Tunisia; CA, Canada; JP, Japan; PS, randomized parallel-group trial; CS, randomized crossover trial; M, male; F, female; CWI, cold water immersion group; CON, control group; NR, not reported.

**Table 2:** Interventional strategies and outcome indicators of the included literature.

Included studies	Exercise plan	Intervention program		Outcome indicator	Monitoring time point	Immersion depth groupings	Adverse events
		CWI	CON				
Sánchez 2018 (36)	8 sets × 30 s vertical jumps,90 s rest between sets	12.0 ± 0.4 °C; 12 min; submerged up to the navel	PR 12 min	CMJ	24;48	WB-CWI	NR
Amir 2017 (37)	10 sets of 10 repetitions CMJ	15.0 ± 1.0 °C; 15 min; Immersion to the iliac crest	PR 15 min	CK; DOMS	24;48;72	LB-CWI	NR
Anderson 2017 (38)	Interval sprinting	C1:5.0 ± 1.0 °C; 12 min; Immersion to the iliac crest; C2:14.0 ± 1.0 °C, 12 min; Immersion to the iliac crest	PR 12 min	CK	0;24;48;72	LB-CWI	NR
Angelopoulos 2022 (39)	Step jumps (5 sets × 20 reps)	10.0 °C; 10 min; Immersion to the anterior superior iliac spine	PR 10 min	DOMS	0;24	LB-CWI	NR
Broatch 2014 (49)	High-intensity interval training	10.3 ± 0.2 °C; 15 min; submerged up to the navel	PR 15 min	DOMS	0;24;48	WB-CWI	NR
Crowther 2017 (4)	High-intensity interval training	15.0 °C; 14 min; Immersed up to the shoulders	PR 14 min	DOMS; CMJ	0;24;48	WB-CWI	NR
Dantas 2019 (40)	10-kilometer run	10.0 °C; 10 min; Immersion to the anterior superior iliac spine	PR 10 min	MVIC	0;24	LB-CWI	NR
Doeringer 2017 (41)	Centrifugal training	10.0 °C; 25 min; Immersion to the iliac crest	PR 25 min	DOMS	24;48	LB-CWI	NR
Elias 2012 (50)	Standardized Australian Football Training	12.0 °C; 14 min; Immerse to the xiphoid process	PR 14 min	CMJ; DOMS; CK	0;24;48	WB-CWI	NR
Fonseca 2016 (51)	Jiu-Jitsu Training	6.0 ± 0.5 °C; 16 min; Immersed up to the neck	PR 16 min	CMJ	0;24;48	WB-CWI	NR
Haq 2022 (42)	30-minute downhill run	15.0 ± 0.5 °C; 10 min; Immersion to the iliac crest	PR 15 min	CK	24	LB-CWI	NR
Heinke 2024 (43)	eccentric exercise	11.0 ± 0.5 °C; 12 min; submerged up to the hips	PR 12 min	DOMS; CK	24;48;72	LB-CWI	NR
Higgins 2013 (44)	Simulated rugby match	10.0–12.0 °C; 10 min; Immersion to the iliac crest	PR 10 min	DOMS; CMJ	0;48;72	LB-CWI	NR
Kim 2023 (52)	eccentric resistance training	8.0 °C; 10 min; Immersion to the iliac crest	PR 10 min	MVIC; DOMS	24;48	LB-CWI	NR
Leeder 2015 (45)	Loughborough Intermittent Shuttle Test, LIST	14.0 °C; 14 min; Seated/standing immersion	PR 14 min	MVIC; CK; DOMS; CMJ	24;48;72	LB-CWI/WB-CWI	NR
Li 2023 (53)	Simulated Basketball Game	5.0 ± 1.0 °C; 12 min; Immersion to the iliac crest	PR 12 min	CMJ	0;24	LB-CWI	NR
Machado 2016 (35)	eccentric resistance training	C1: 9.0 ± 1.0 °C; 15 min; Immersion to the iliac crest; C2: 14.0 ± 1.0 °C; 15minImmersion to the iliac crest	PR 15 min	MVIC; CK; DOMS	0;24;48;72	LB-CWI	NR
Minett 2013 (54)	High-intensity interval training	10.0 ± 0.4 °C; 20 min; submerged up to the sternum	PR 20 min	CK	0;24	WB-CWI	NR
Nasser 2023 (55)	Loughborough Intermittent Shuttle Test, LIST	11.3 ± 0.2 °C; 15 min; submerged up to the sternum	PR 15 min	CMJ	24;48	WB-CWI	NR
Ottone 2022 (56)	eccentric resistance training	15 °C; 15 min; Immerse to the xiphoid process	PR 15 min	CK	0;24	WB-CWI	NR
Pesenti 2020 (34)	Resistance Training	10 °C; 10 min; Immersion to the iliac crest	PR 10 min	MVIC; DOMS	24;48;72	LB-CWI	NR
Pointon 2012 (57)	High-intensity interval training	9.2 ± 0.2 °C; 18 min; Immersion to the iliac crest	PR 18 min	CK	0;24	LB-CWI	NR
Rupp 2012 (46)	Yo-Yo Intermittent Recovery Test	12.0 °C; 15 min; submerged up to the navel	PR 15 min	CMJ	24;48	WB-CWI	NR
Silva 2017 (58)	Simulated Jiu-Jitsu Competition	12.0 °C;6 min; submerged up to the sternum	PR 6 min	CK	0	WB-CWI	NR
Takeda 2014 (61)	80-Minute Rugby Simulation Training	15.0 °C; 10 min; Full-body immersion	PR 10 min	DOMS; CMJ	24	WB-CWI	NR
Valle 2008 (59)	Centrifugal Resistance Training	15.0 °C; 14 min; Immersed up to the shoulders	PR 14 min	MVIC; CK	24;48;72	WB-CWI	NR
Wei 2025 (3)	Lower-body eccentric exercise	11.0–15.0 °C; 12 min; Immersion to the iliac crest	PR 12 min	CK	24;48;72	LB-CWI	NR
White 2014 (60)	High-intensity interval training	10.0 °C; 10 min; Immersion to the iliac crest	PR 10 min	DOMS	0;24;48	LB-CWI	NR
Wiewelhove 2018 (47)	Half Marathon (21.1 km)	15.0; 15 min; Immersion to the iliac crest	PR 15 min	CMJ; DOMS; CK	0;24	LB-CWI	NR
Yoshimura 2023 (48)	Centrifugal training	20.0 °C; 20 min; Immersion to the iliac crest	PR 20 min	MVIC; CMJ; DOMS	48	LB-CWI	NR

CWI, cold water immersion group; CON, control group; PR, seated passive recovery; MVIC, maximum voluntary isometric contraction; CMJ, countermovement jump; CK, creatine kinase; DOMS, delayed onset muscle soreness; WB-CWI, whole-body cold-water immersion (water level above the iliac crest, including navel, sternum, or shoulder level); LB-CWI, lower-body cold-water immersion (water level at or below the iliac crest/hip level); 0, immediately post-intervention; 24, 24 h post-exercise; 48, 48 h post-exercise; 72, 72 h post-exercise; NR, Not reported.

### 3.3 Quality assessment of included literature

**Figure 3:**
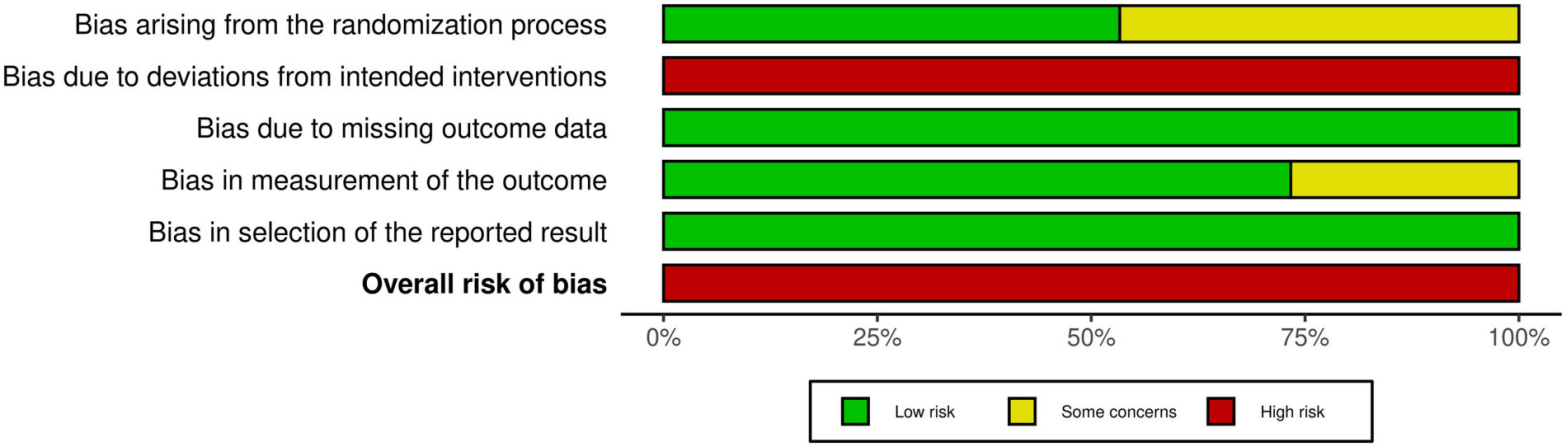
Risk of bias graph for included studies.

**Figure 4:**
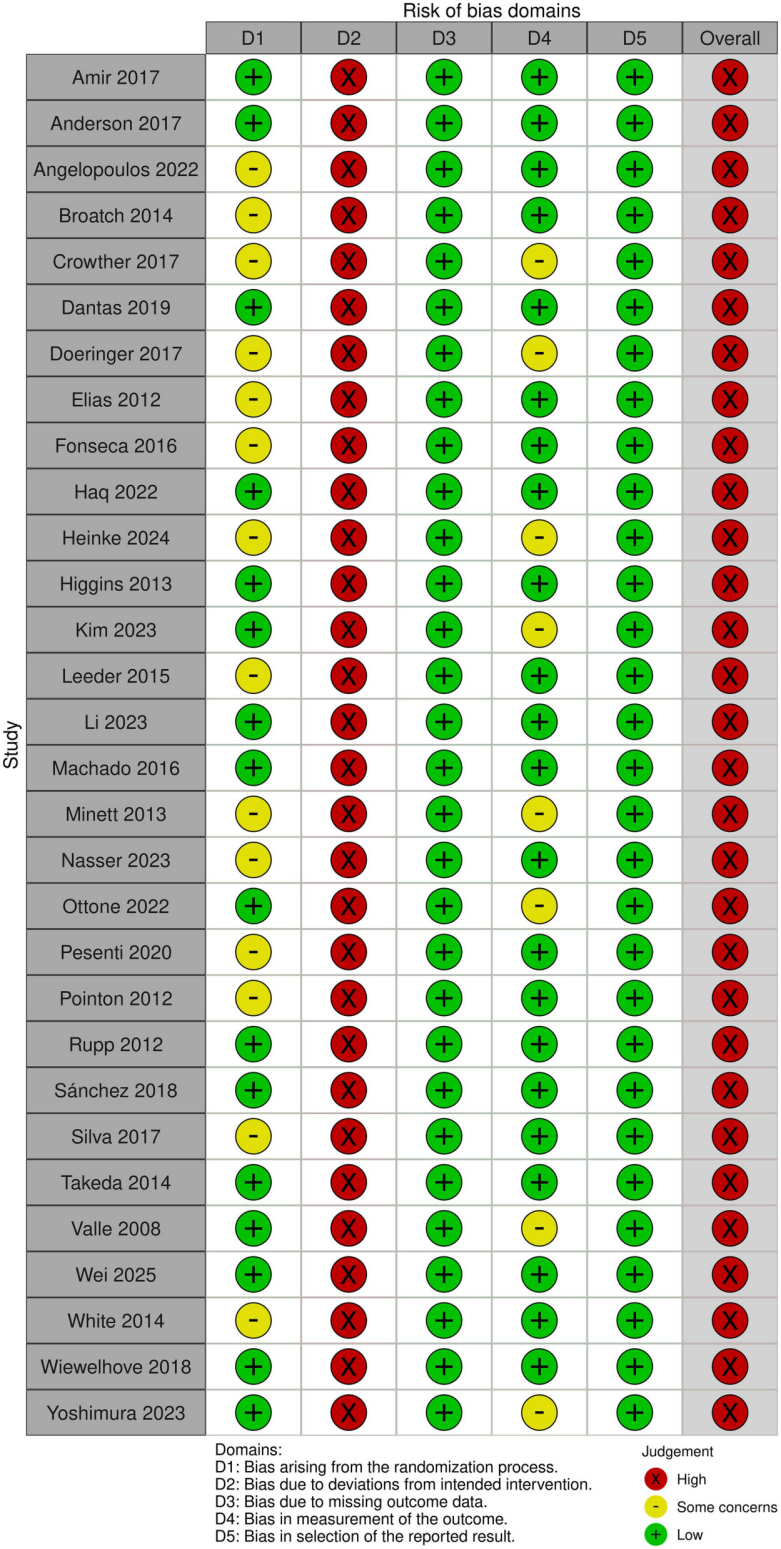
Summary of bias risk in included studies.

While the RoB 2 assessment indicated a high overall risk of bias due to the infeasibility of blinding participants (Domain 2), the structural quality of the included studies remained high. All studies showed low risk regarding data completeness and selective reporting, and the majority (approx. 75%) employed blinded assessors to mitigate detection bias. Consistent with this, PEDro scores of 6–8 confirmed a generally high methodological standard. Therefore, despite the unavoidable limitation of performance bias, the synthesized evidence is considered reliable (Figures 3, 4; Table 3).

**Table 3:** Quality assessment of included literature.

Study	1	2	3	4	5	6	7	8	9	10	11	Total score
Sánchez 2018	1	1	0	1	0	0	0	1	1	1	1	6
Amir 2017	1	1	0	1	0	0	0	1	1	1	1	6
Anderson 2017	1	1	0	1	0	0	0	1	1	1	1	6
Angelopoulos 2022	1	1	0	1	0	0	1	1	1	1	1	7
Broatch 2014	1	1	0	1	0	0	1	1	1	1	1	7
Crowther 2017	1	1	0	1	0	0	0	1	1	1	1	6
Dantas 2019	1	1	0	1	0	0	1	1	1	1	1	7
Doeringer 2017	1	1	0	1	0	0	0	1	1	1	1	6
Elias 2012	1	1	0	1	0	0	0	1	1	1	1	6
Fonseca 2016	1	1	0	1	0	0	0	1	1	1	1	6
Haq 2022	1	1	0	1	0	0	0	1	1	1	1	6
Heinke 2024	1	1	0	1	0	0	0	1	1	1	1	6
Higgins 2013	1	1	0	1	0	0	0	1	1	1	1	6
Kim 2023	1	1	1	1	0	0	0	1	1	1	1	7
Leeder 2015	1	1	0	1	0	0	0	1	1	1	1	6
Li 2023	1	1	0	1	0	0	0	1	1	1	1	6
Machado 2016	1	1	1	1	0	0	1	1	1	1	1	8
Minett 2013	1	1	0	1	0	0	0	1	1	1	1	6
Nasser 2023	1	1	0	1	0	0	0	1	1	1	1	6
Ottone 2022	1	1	0	1	0	0	0	1	1	1	1	6
Pesenti 2020	1	1	0	1	0	0	0	1	1	1	1	6
Pointon 2012	1	1	0	1	0	0	0	1	1	1	1	6
Rupp 2012	1	1	0	1	0	0	0	1	1	1	1	6
Silva 2017	1	1	0	1	0	0	0	1	1	1	1	6
Takeda 2014	1	1	0	1	0	0	0	1	1	1	1	6
Valle 2008	1	1	0	1	0	0	0	1	1	1	1	6
Wei 2025	1	1	1	1	0	0	1	1	1	1	1	8
White 2014	1	1	0	1	0	0	0	1	1	1	1	6
Wiewelhove 2018	1	1	0	1	0	0	0	1	1	1	1	6
Yoshimura 2023	1	1	0	1	0	0	0	1	1	1	1	6

1 denotes subject eligibility; 2 denotes random allocation; 3 denotes allocation concealment; 4 denotes baseline similarity; 5 denotes subject blinding; 6 denotes clinician blinding; 7 denotes assessor blinding; 8 denotes dropout rate <15%; 9 denotes intention-to-treat analysis; 10 denotes between-group statistical analysis; 11 denotes point measurements and difference values.

### 3.4 Meta-analysis results

#### 3.4.1 CK

**Figure 5:**
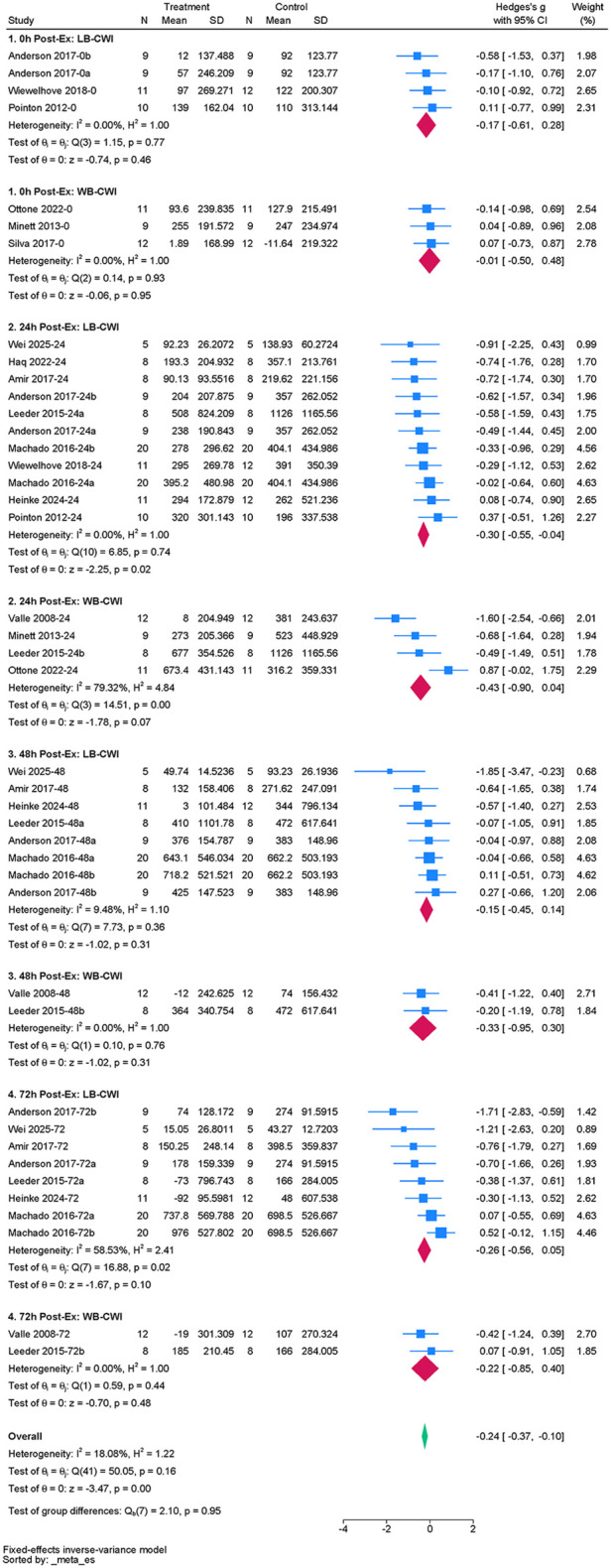
Forest plot of the effects of CWI on CK stratified by follow-up time points and body regions.

Fourteen studies were included to assess the effect of acute CWI on post-exercise CK levels (3, 35, 37, 38, 42, 43, 45, 47, 50, 54, 56–59). The overall pooled analysis showed low heterogeneity among the studies (I^2^ = 18.08%, *P* = 0.16). The fixed-effect model showed that CWI significantly reduced post-exercise CK levels compared to the control group. The overall effect size was small (g = –0.24; 95% CI: −0.37 to −0.10, *P* < 0.01) (Figure 5). Stratified subgroup analysis examined follow-up time points and immersion regions. Partial CWI showed a significant effect in reducing CK levels compared to the control group only at 24 h post-exercise (g = –0.30, *P* = 0.02). The effect of whole-body CWI did not reach statistical significance (*P* > 0.05). However, there was no statistical difference between partial and whole-body CWI in reducing CK levels (*P_m_* > 0.05) (Table 4).

**Table 4:** Subgroup analysis of the effects of CWI on CK by body region and follow-up time points.

Time point	Immersion site	K	Hedges' g (95% CI)	*P_d_*	I^2^ (%)	*P_m_*
0 h	LB-CWI	4	−0.17[−0.61, 0.28]	0.46	0.00	0.648
	WB-CWI	3	−0.01[−0.50, 0.48]	0.95	0.00	
24 h	LB-CWI	11	−0.30[−0.55, −0.04]	0.02	0.00	0.626
	WB-CWI	4	−0.43[−0.90, 0.04]	0.07	79.32	
48 h	LB-CWI	8	−0.15[−0.45, 0.14]	0.31	9.48	0.629
	WB-CWI	2	−0.33[−0.95, 0.30]	0.31	0.00	
72 h	LB-CWI	8	−0.26[−0.56, 0.05]	0.10	58.53	0.924
	WB-CWI	2	−0.22[−0.85, 0.40]	0.48	0.00	

K, number of included studies; P_d_, *p*-value of the pooled effect size for the subgroup; I^2^(%), statistics for heterogeneity; P_m_, *p*-value for the test of subgroup differences.

#### 3.4.2 DOMS

**Figure 6:**
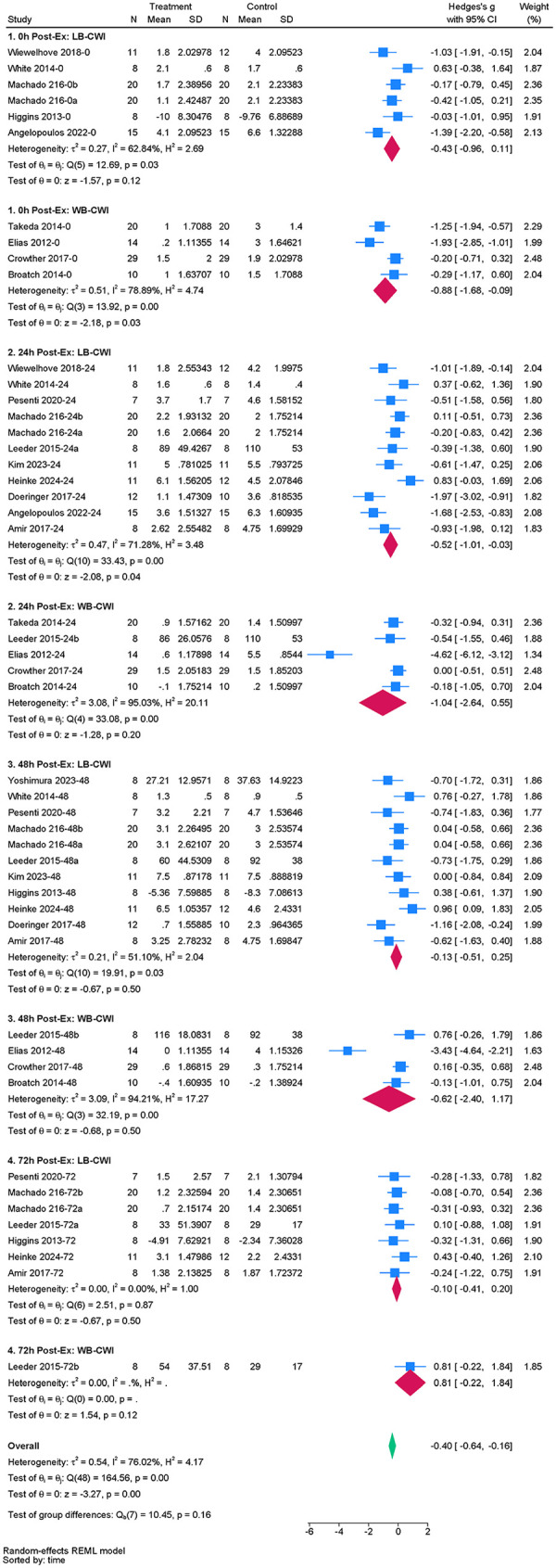
Forest plot of the effects of CWI on DOMS stratified by follow-up time points and body regions.

The review included 16 studies to evaluate the effect of acute CWI on post-exercise DOMS (4, 34, 35, 37, 39, 41, 43–45, 47–50, 52, 60, 61). The overall pooled analysis revealed high heterogeneity among the studies (I^2^ = 76.02%, *P* = 0.00). The random-effects model indicated that CWI significantly alleviated the degree of post-exercise DOMS compared to the control group. The overall effect size was small to moderate (g = –0.40; 95% CI: −0.64 to −0.16, *P* < 0.01) (Figure 6). Subgroup analysis showed that whole-body CWI significantly alleviated post-exercise DOMS at 0 h compared to the control group (g = –0.88, *P* = 0.03). Partial CWI had no significant effect at this time point (*P* > 0.05). Conversely, partial CWI significantly alleviated DOMS at 24 h (g = –0.52, *P* = 0.04), while whole-body CWI had no significant effect (*P* > 0.05). However, tests between subgroups found no significant difference between whole-body and partial CWI in reducing post-exercise DOMS (*P_m_* > 0.05) (Table 5).

**Table 5:** Subgroup analysis of the effects of CWI on DOMS by body region and follow-up time points.

Time point	Immersion site	K	Hedges' g (95% CI)	*P_d_*	I^2^ (%)	*P_m_*
0 h	LB-CWI	6	−0.43 [−0.96, −0.11]	0.12	62.84	0.229
	WB-CWI	4	−0.88 [−1.68, −0.09]	0.03	78.89	
24 h	LB-CWI	11	−0.52 [−1.01, −0.03]	0.04	71.28	0.867
	WB-CWI	5	−1.04 [−2.64, 0.55]	0.20	95.03	
48 h	LB-CWI	11	−0.13 [−0.51, 0.25]	0.50	51.10	0.751
	WB-CWI	4	−0.62 [−2.40, 1.17]	0.50	94.21	
72 h	LB-CWI	7	−0.10 [−0.41, 0.20]	0.50	0.00	0.095
	WB-CWI	1	0.81 [−0.22, 1.84]	0.12	NA	

K, number of included studies; *P_d_*, *p*-value of the pooled effect size for the subgroup; I^2^(%), statistics for heterogeneity; P_m_, *p*-value for the test of subgroup differences; N/A, stands for not applicable.

#### 3.4.3 CMJ

**Figure 7:**
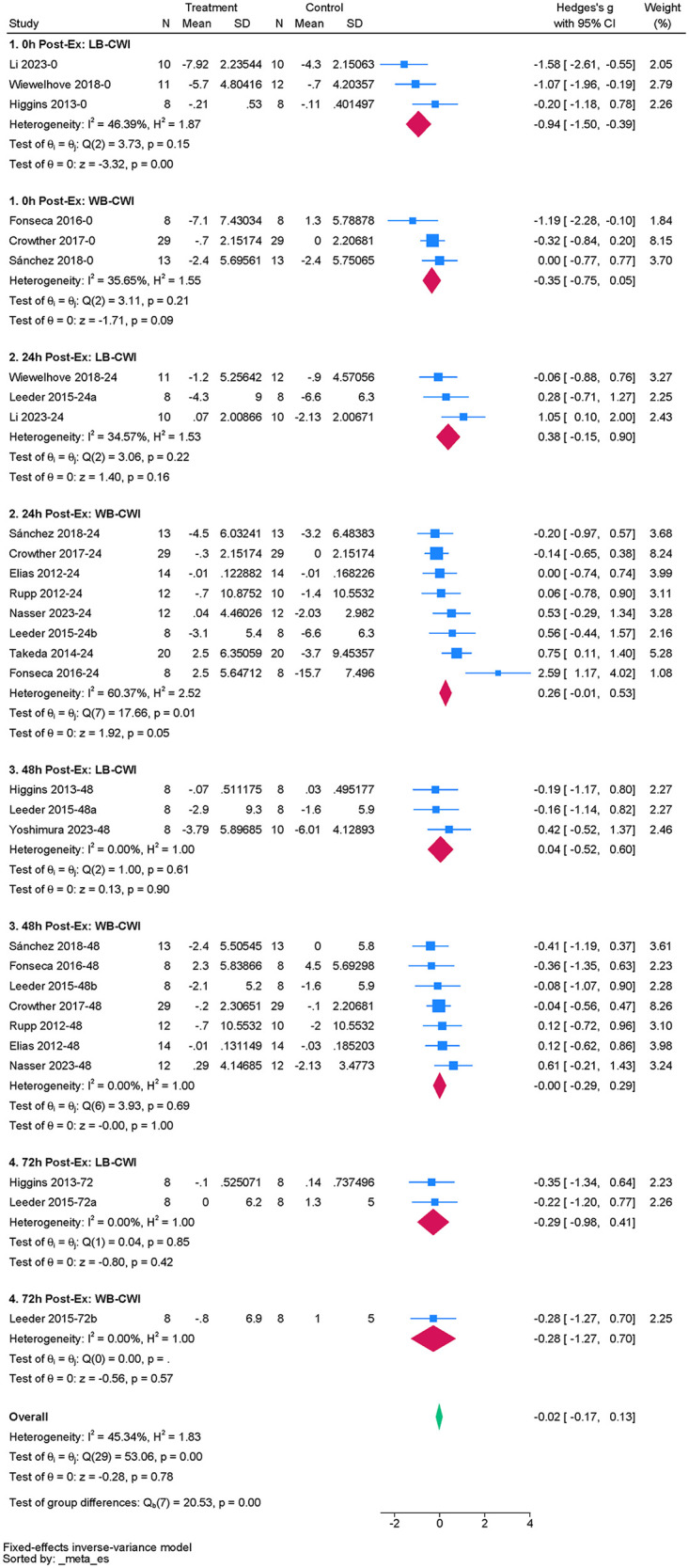
Forest plot of the effects of CWI on CMJ stratified by follow-up time points and body regions.

The analysis included 12 studies to assess the effect of acute CWI on post-exercise CMJ (4, 36, 44–48, 50, 51, 53, 55, 61). The overall pooled analysis showed moderate heterogeneity among the studies (I^2^ = 45.34%, *P* < 0.01). The fixed-effect model showed that CWI did not significantly alter post-exercise CMJ performance compared to the control group (g = –0.02; 95% CI: −0.17 to 0.13, *P* > 0.05) (Figure 7). Subgroup analysis indicated that partial CWI significantly inhibited CMJ performance only at 0 h compared to the control group. This negative effect was large (g = –0.94, *P* < 0.01). Whole-body CWI showed no significant effect (*P* = 0.09). However, tests between subgroups revealed no statistical difference between whole-body and partial CWI regarding their impact on post-exercise CMJ performance (*P_m_* > 0.05). No significant effects were observed at other follow-up time points (*P* > 0.05) (Table 6).

**Table 6:** Subgroup analysis of the effects of CWI on CMJ by body region and follow-up time points.

Time point	Immersion site	K	Hedges' g (95% CI)	*P_d_*	I^2^ (%)	*P_m_*
0 h	LB-CWI	3	−0.94 [−1.50, −0.39]	0.00	46.39	0.090
	WB-CWI	3	−0.35 [−0.75, 0.05]	0.09	35.65	
24 h	LB-CWI	3	0.38 [−0.15, 0.90]	0.16	34.57	0.702
	WB-CWI	8	0.26 [−0.01, 0.53]	0.05	60.37	
48 h	LB-CWI	3	0.04 [−0.52, 0.60]	0.90	0.00	0.906
	WB-CWI	7	−0.00 [−0.29, 0.29]	1.00	0.00	
72 h	LB-CWI	2	−0.29 [−0.98, 0.41]	0.50	0.00	0.996
	WB-CWI	1	−0.28 [−1.27, 0.70]	0.57	NA	

K, number of included studies; *P_d_*, *p*-value of the pooled effect size for the subgroup; I^2^(%), statistics for heterogeneity; *P_m_*, *p*-value for the test of subgroup differences; N/A, stands for not applicable; NR, not reported.

#### 3.4.4 MVIC

**Figure 8:**
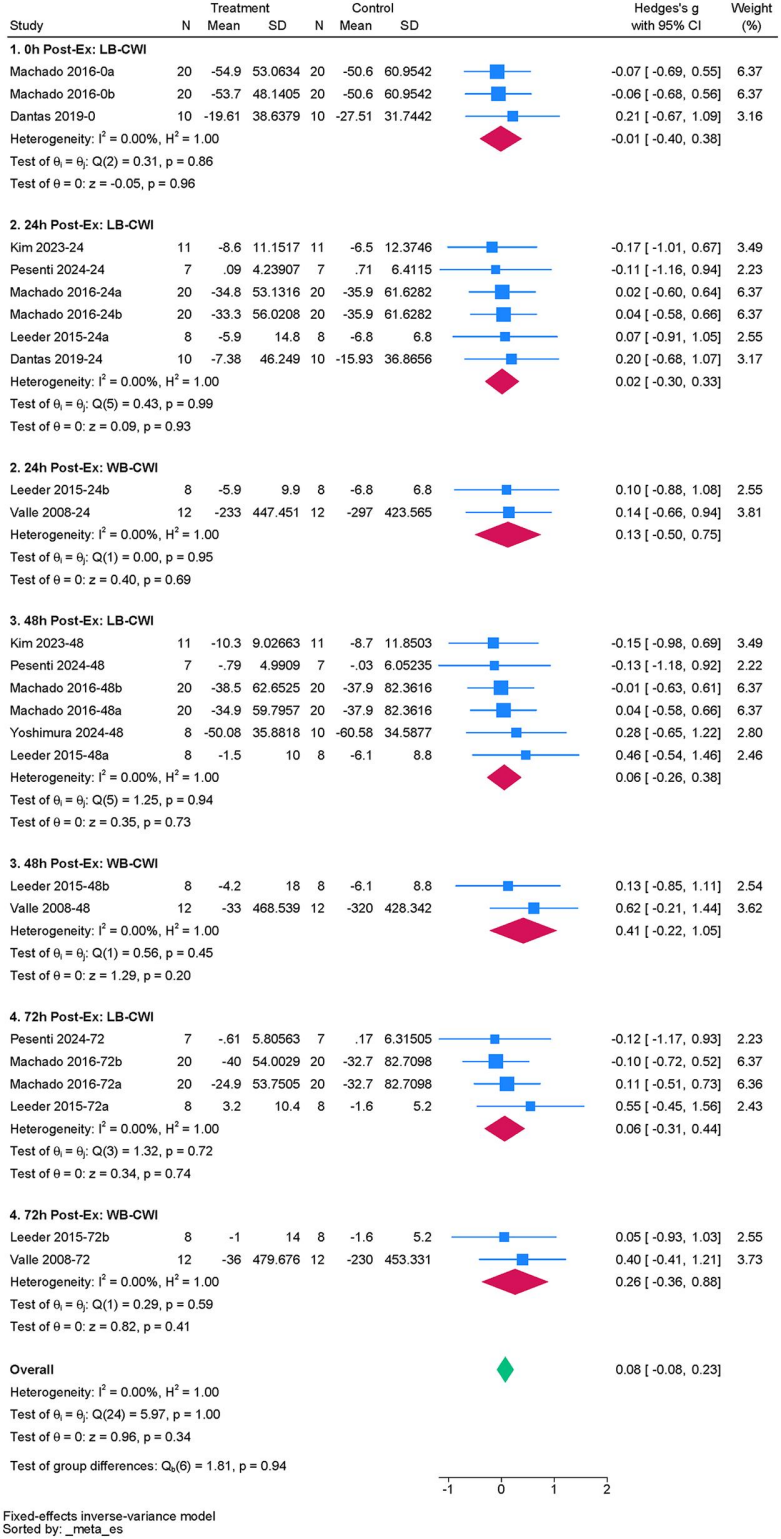
Forest plot of the effects of CWI on MVIC stratified by follow-up time points and body regions.

The study included seven studies to assess the effect of acute CWI on post-exercise MVIC (34, 35, 40, 45, 48, 52, 59). The overall pooled analysis showed no statistical heterogeneity among the studies (I^2^ = 0.00%, *P* = 1.00). The fixed-effect model indicated that CWI did not significantly affect post-exercise MVIC performance compared to the control group (g = 0.08; 95% CI: −0.08 to 0.23, *P* > 0.05) (Figure 8). Subgroup analysis showed that neither whole-body nor partial CWI significantly altered post-exercise MVIC performance at any follow-up time point (all *P* > 0.05). Furthermore, no significant difference was observed between whole-body and partial CWI regarding the recovery of post-exercise MVIC (*Pm* > 0.05) (Table 7).

**Figure 9:**
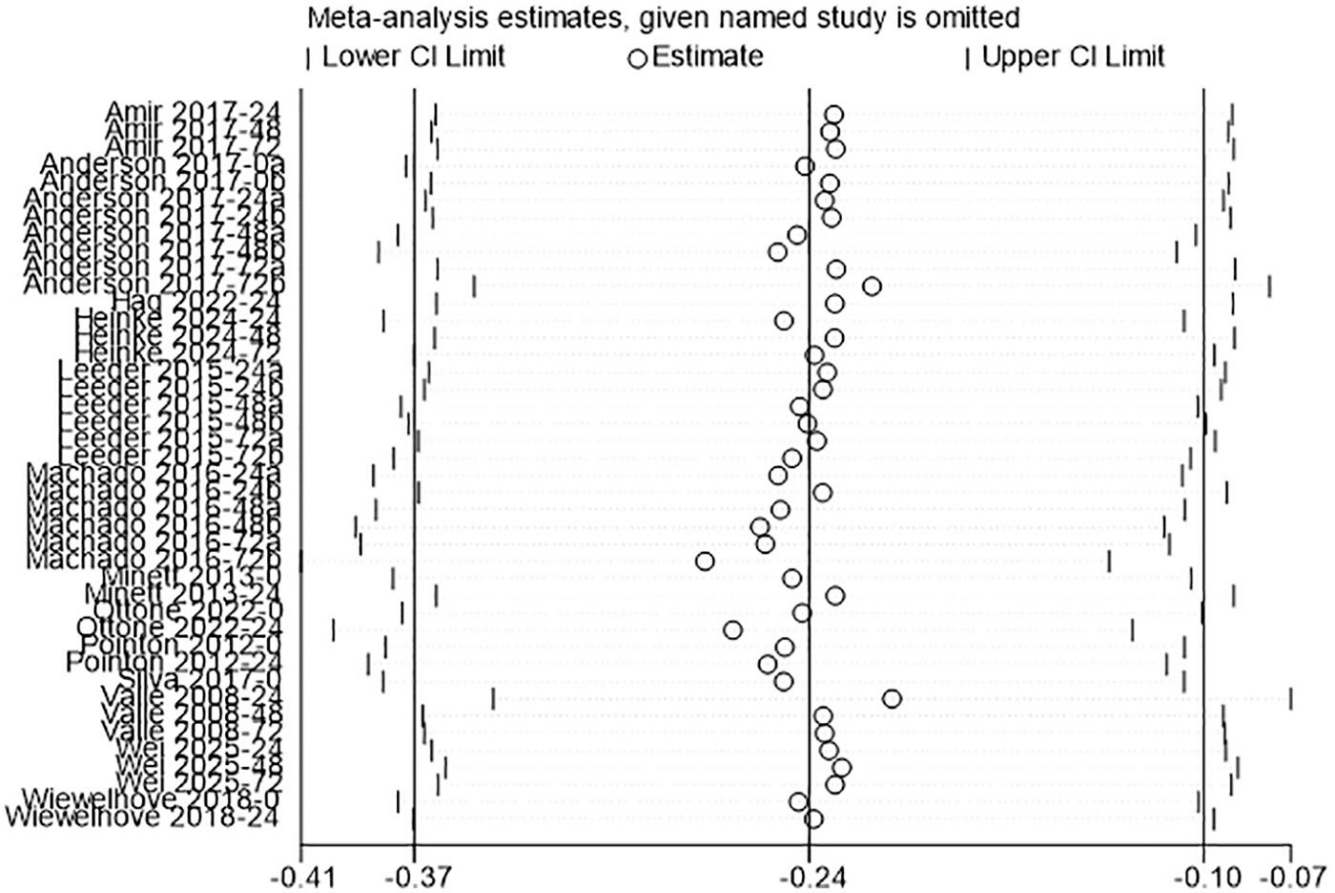
Sensitivity analysis for CK.

**Figure 10:**
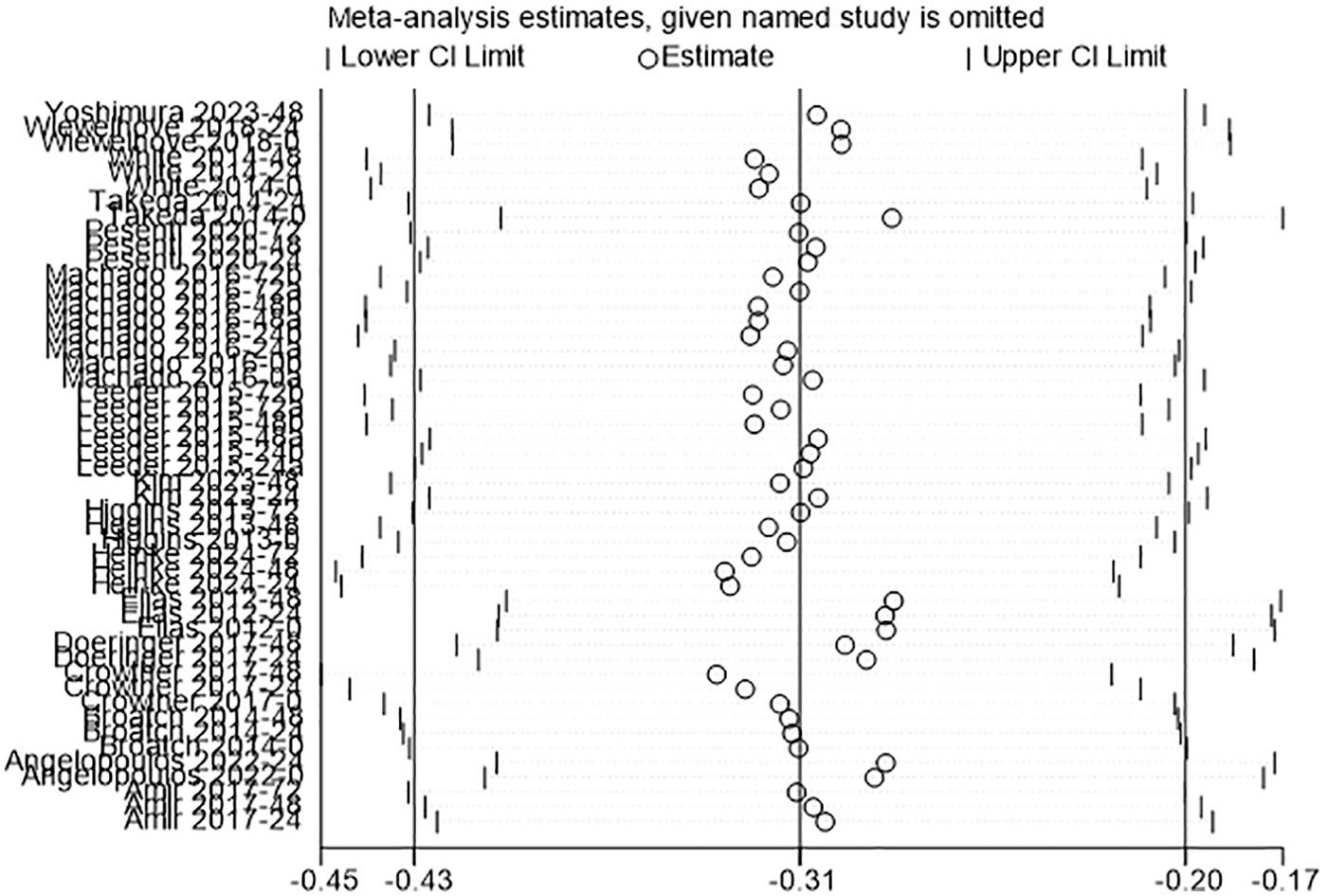
Sensitivity analysis for DOMS.

**Figure 11:**
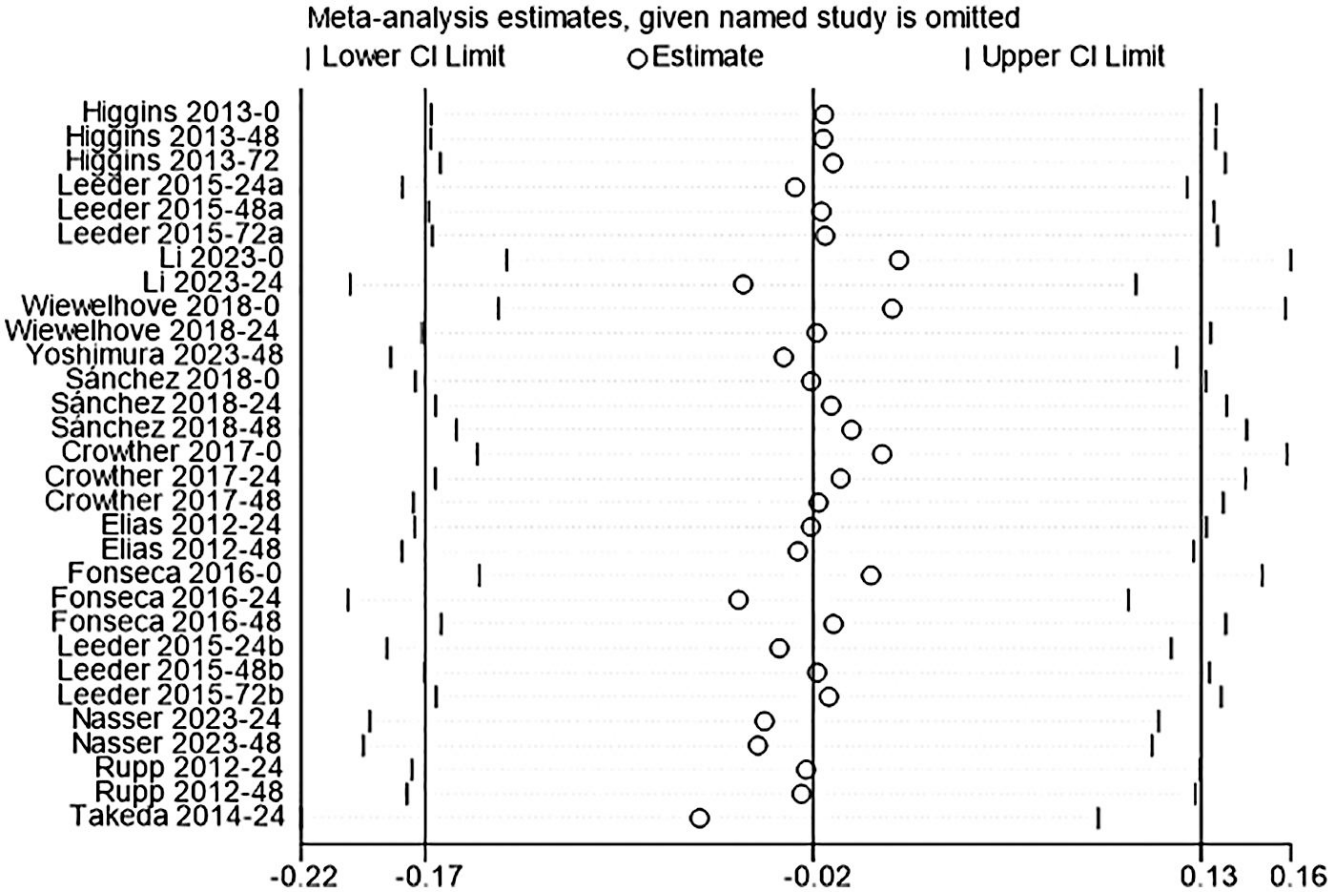
Sensitivity analysis for CMJ.

**Figure 12:**
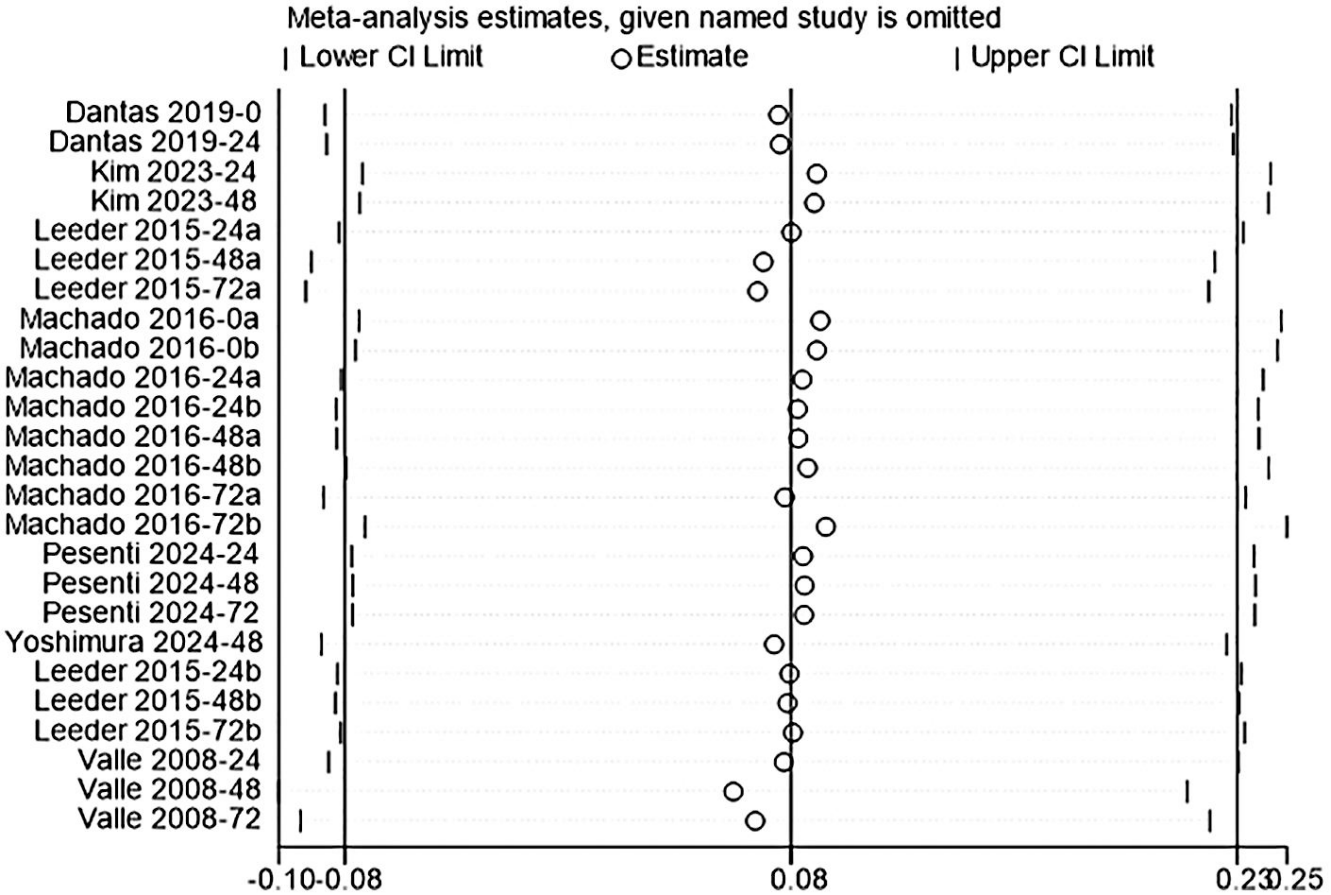
Sensitivity analysis for MVIC.

**Figure 13:**
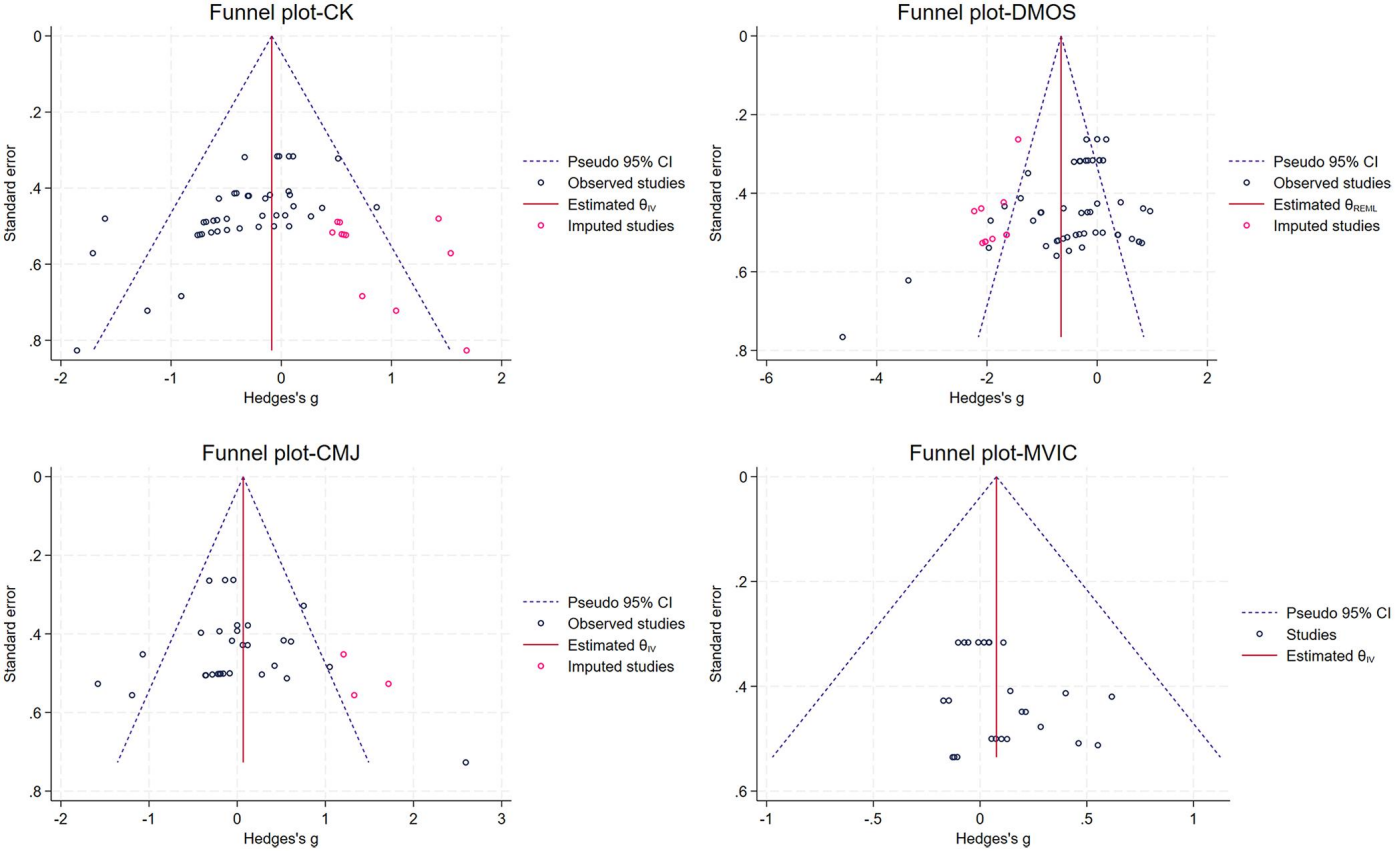
Funnel plots of publication bias for all included outcomes.

**Table 7:** Subgroup analysis of the effects of CWI on CMJ by body region and follow-up time points.

Time point	Immersion site	K	Hedges' g (95% CI)	*P_d_*	I^2^ (%)	*P_m_*
0 h	LB-CWI	3	−0.01[−0.40, 0.38]	0.96	0.00	NA
	WB-CWI	NR				
24 h	LB-CWI	6	0.02[−0.30, 0.33]	0.93	0.00	0.757
	WB-CWI	2	0.13[−0.50, 0.75]	0.69	0.00	
48 h	LB-CWI	6	0.06[−0.26, 0.38]	0.73	0.00	0.321
	WB-CWI	2	0.41[−0.22, 1.05]	0.20	0.00	
72 h	LB-CWI	4	0.06[−0.31, 0.44]	0.74	0.00	0.598
	WB-CWI	2	0.26[−0.36, 0.88]	0.41	0.00	

K, number of included studies; *P_d_*, *p*-value of the pooled effect size for the subgroup; I^2^(%), statistics for heterogeneity; *P_m_*, *p*-value for the test of subgroup differences; N/A, stands for not applicable.

#### 3.4.5 Sensitivity analysis

Sensitivity analysis using the leave-one-out method confirmed the robustness of the study results. For all outcome measures (CK, DOMS, CMJ, MVIC), the removal of any single study did not alter the direction or statistical significance of the pooled effects. The re-tested 95% confidence intervals remained highly consistent with the original results. This indicated that the conclusions were not driven by any single study (Figures 9–12).

Sensitivity analysis identified the study by Elias (2012) as the primary source of the significant heterogeneity observed for DOMS (I^2^ = 76.02%) (50). This study reported an extremely large effect size (g = –4.62). This likely resulted from the use of professional Australian football players as subjects. Additionally, the study employed high-intensity small-sided games as the inducement method. These factors led to higher baseline muscle damage and amplified the intervention effect. The exclusion of this study significantly reduced heterogeneity to a moderate level (I^2^ = 53.71%). The direction and significance of the pooled effect size remained unchanged. This result further confirmed the reliability of the meta-analysis.

#### 3.4.6 Publication bias

Visual inspection of the funnel plots revealed a generally symmetrical distribution for most outcomes. The absence of significant publication bias for CMJ (*P* = 0.678) and MVIC (*P* = 0.213) was further confirmed by Egger's regression tests. However, a potential risk of publication bias was indicated for CK (*P* < 0.001) and DOMS (*P* = 0.025).

The trim-and-fill method was employed to assess result robustness for these two indicators. For CK, the adjusted Hedges' g shifted from the original −0.236 (95% CI: −0.369 to −0.103) to −0.086 (95% CI: −0.210 to 0.037). The subsequent loss of statistical significance suggested that the original CK findings might have been overestimated due to publication bias. Consequently, these results should be interpreted with caution. Regarding DOMS, the Hedges' g was adjusted from −0.412 (95% CI: −0.696 to −0.128) to −0.172 (95% CI: −0.986 to −0.438) after correction. Although the effect size was altered, the result remained statistically significant. This demonstrated the robustness of the findings for DOMS.

In summary, no publication bias was detected for CMJ and MVIC. While the DOMS results were influenced by bias, they remained robust following adjustment. In contrast, the CK results were substantially affected by publication bias and warrant cautious interpretation (Figure 13).

## 4 Discussion

### 4.1 Main findings

The acute and delayed effects of CWI at different body regions (whole-body vs. partial) on post-exercise muscle damage recovery were quantitatively evaluated through a systematic review and meta-analysis. The main findings were as follows: First, post-exercise CK levels were significantly reduced and DOMS was alleviated by CWI compared to seated rest. However, the restorative effect on neuromuscular function was limited. No significant improvement in MVIC was observed. A significant inhibitory effect on immediate post-exercise CMJ performance was shown. Notably, while the results for DOMS were robust, statistical significance for CK disappeared after adjustment using the trim-and-fill method. This suggested that its efficacy might have been overestimated due to publication bias. Second, subgroup analysis focusing on the “anatomical dimension” confirmed no statistical difference between whole-body and partial immersion across all outcome measures (*P_m_* > 0.05). This result revised the traditional hypothesis based on hydrodynamic (hydrostatic pressure) and neurophysiological differences mentioned in the Introduction (19). It indicated that recovery benefits meeting the physiological threshold are generated as long as the immersion range covers the affected muscle groups. Whole-body immersion is not required. Finally, the benefits of CWI showed significant time-dependency. They were primarily concentrated within the 24-hour post-exercise window. Conversely, immediate post-exercise partial cold therapy showed significant inhibition of explosive power. This suggested that caution regarding the temporary impairment of neuromuscular function is needed during acute application.

### 4.2 Effects on muscle damage

A significant efficacy of CWI in reducing post-exercise CK levels was confirmed by this study. This is consistent with previous research findings (36, 63). CK is widely regarded clinically as a key marker for monitoring skeletal muscle damage. Elevated levels are typically associated with muscle cell membrane damage and myofiber leakage induced by strenuous exercise (64, 65). Mechanistically, the increase in membrane permeability is inhibited by single acute CWI through the rapid reduction of muscle temperature. Consequently, CK leakage into the blood is reduced (17). Simultaneously, physical pressure is exerted on the muscle by the hydrostatic pressure effect of immersion. Cell swelling is limited and metabolic waste clearance is promoted. The structural integrity of muscle fibers is further protected (60). Furthermore, the infiltration and diffusion of inflammatory cells and pro-inflammatory mediators (such as IL-6) are effectively inhibited by inducing local vasoconstriction. Acute inflammatory responses and tissue edema are alleviated. An optimal environment for muscle regeneration is created (37, 66). Subsequently, blood redistribution is facilitated during the reperfusion phase following vasoconstriction. Metabolic product clearance is accelerated and reoxygenation of affected tissues is improved. Nutrient and oxygen supply to the muscle is increased by enhanced vasodilation. The repair process is accelerated and normal physiological function is restored (67). Additionally, oxidative stress is triggered by strenuous exercise. Free radical generation is accelerated, exacerbating muscle damage (51). However, the activity of antioxidant enzymes such as superoxide dismutase and glutathione peroxidase is significantly enhanced by cold therapy. The clearance of excess reactive oxygen species is accelerated. Damage to muscle cells from oxidative stress is mitigated (54).

However, no significant difference was found between whole-body and partial CWI in reducing CK levels. This may be primarily because lower-limb dominant exercise protocols, such as plyometric jumps or shuttle runs, were employed in some of the included literature (37, 39, 40, 47). EIMD was mainly concentrated in muscle groups such as the quadriceps and triceps sura. Complete immersion of the affected target tissues was achieved by partial immersion with water levels up to the iliac crest. Local metabolic inhibition was sufficiently induced via transcutaneous thermal conduction (68). Although a larger central blood volume shift is indeed induced by whole-body immersion, the pressure gradient required to counteract gravitational edema and promote tissue fluid return to the lymphatic system is sufficiently generated by the hydrostatic pressure exerted on the lower limbs during partial immersion (69). Therefore, a “saturation effect” was produced by partial immersion for lower limb muscle damage. Further expansion of the immersion area did not translate into additional biochemical clearance benefits. Moreover, excessive systemic stress associated with whole-body cold exposure was avoided (70).

### 4.3 Effects on muscle soreness

Significant and relatively robust efficacy of CWI in alleviating post-exercise DOMS was confirmed. It was consistently indicated by previous randomized controlled trials and systematic reviews that pain thresholds are increased and subjective pain sensation is reduced after CWI intervention (13, 26). DOMS typically peaks 24–72 h post-exercise. Its mechanism is primarily related to mechanical muscle fiber damage and the subsequently induced local inflammatory response and metabolite accumulation (71). It is commonly assessed clinically using Visual Analog Scale scores (72). The analgesic mechanism of CWI involves complex physiological and psychological multidimensional interactions. Local metabolic rate and oxygen consumption are reduced by vasoconstriction and tissue temperature decrease caused by cold stimulation. The accumulation of metabolites (such as lactate, prostaglandins, bradykinin) is reduced. Consequently, chemical pain stimulation is alleviated (26). Exudation of inflammatory cells and release of cytokines (IL-6, TNF-α, IL-1β) are inhibited by low temperature. Secondary tissue damage and pain signal transmission are limited (60). Most importantly, nerve conduction velocity is reduced and pain threshold is increased by cold stimulation, thereby directly relieving local pain. Tissue fluid return is promoted and edema and mechanical tenderness are reduced by hydrostatic pressure effects and hemodynamic changes (73). Furthermore, the analgesic effect of CWI stems partly from the immediate improvement of subjective recovery and emotional state. This subjective and emotional improvement is associated with sympathetic activation induced by cold exposure. It is manifested as increased plasma norepinephrine/epinephrine levels. Alertness and vitality are enhanced, producing a positive placebo effect (74, 75).

However, a clear time-dependent decay is presented by this analgesic effect. The analgesic effect of a single CWI session (partial or whole-body) tended to disappear 48 h post-exercise. This indicated that its residual effect is extremely limited. Physiologically, this period corresponds to the peak active period of muscle damage repair and the inflammatory cascade. Cytokine signaling and pain drivers are at a high stage at this time (76). Klich et al. further elucidated the distinction between acute and chronic alterations; they demonstrated that while CWI induced immediate improvements in pressure pain thresholds (PPT), these analgesic benefits did not uniformly persist into the delayed recovery phase, particularly across muscle groups with distinct fatigue characteristics (77). Consequently, the positive effects induced by a single acute CWI session are insufficient to counteract this continuous and complex physiological process over the long term (78). This finding suggests that the optimal window for a single cold therapy session is limited to the acute phase. If efficacy needs to be prolonged, a single intervention is clearly insufficient. Repeated immersion or combination with other recovery strategies needs to be explored in the future to alter the long-term repair trajectory (5, 79).

### 4.4 Effects on muscle strength recovery

MVIC and CMJ are commonly used muscle strength recovery assessment indicators in sports science. They represent the recovery of maximal strength and explosive power, respectively (80, 81). It was shown in this paper that post-exercise MVIC of affected muscle groups was neither significantly improved nor attenuated by CWI. No significant difference was found at other follow-up time points. This result is consistent with the randomized controlled trial by Lee et al. (82). The recovery trajectory of MVIC was not altered by either whole-body or lower-body CWI. This indicates that the analgesic effect (perceptual recovery) of CWI is not synchronously translated into immediate functional improvement (83). The reason may be that low temperature cannot reverse excitation-contraction coupling impairment caused by ultrastructural damage to muscle fibers (such as Z-line disruption) in a short time (57). In contrast, a significant inhibitory effect on explosive power was produced by immediate CWI. The meta-analysis by Moore et al. also confirmed that dynamic/rate-dependent strength (such as CMJ, rate of force development, RTD) is more sensitive to CWI than static peak force (MVIC) (5).

Physiologically, tissue temperature is sharply reduced after acute cold therapy. Nerve conduction velocity (NCV) and motor unit firing rate are slowed. Muscle fiber contraction kinetics are altered. Consequently, RTD and instantaneous explosive power are impaired (84, 85). Empirical evidence from Dixon et al. (86) found that the short-term adverse effects of CWI on CMJ could be partially reversed by increasing muscle temperature through dynamic warm-up. This suggests that immediate performance impairment is closely related to acute changes in muscle temperature and nerve conduction velocity (87). Notably, subgroup analysis further confirmed no difference in functional impact between whole-body and partial immersion. As long as the immersion range covers the main working muscle groups, a cooling effect reaching the inhibition threshold is sufficiently induced by partial CWI (73). Therefore, caution should be exercised with both whole-body and partial immersion during rest periods involving explosive power output (12). Alternatively, sufficient active rewarming strategies must be combined to counteract the aforementioned physiological inhibition.

### 4.5 Limitations

Although a rigorous systematic review process was followed, several limitations must be considered. First is the risk of bias. Due to the nature of CWI, blinding of subjects was impossible. This may introduce psychological expectations or placebo effects. The efficacy of subjective indicators (DOMS) may be particularly overestimated. Moreover, sensitivity analysis suggested potential publication bias for CK results. A conservative attitude towards its true effect size is required. Second is the high heterogeneity of DOMS (I^2^ = 76.02%). Although it was reduced to a moderate level after excluding outlier studies, “multivariable meta-regression” could not be employed to further control for the interactive effects of covariates such as water temperature, duration, and exercise type due to the limited number of studies included in each subgroup. Third is taxonomic ambiguity. Although strict classification criteria based on anatomical landmarks (iliac crest) were established, studies with water levels ranging from the xiphoid process to the neck were still included in the “whole-body immersion” group. These subtle differences in anatomical coverage may have diluted the inter-group effects. Finally, the vast majority of included subjects were male athletes. Given the physiological differences in body fat distribution and thermoregulatory responses in women, the applicability of the conclusions to the female population remains to be verified. Furthermore, this study focused only on acute recovery (≤72 h). The potential interference effects of CWI on long-term training adaptations (such as muscle hypertrophy signaling pathways) were not discussed.

Based on this, future research should focus on: (1) Filling the gender data gap. Research targeting the female population is urgently needed to clarify the potential impact of the menstrual cycle on cold therapy efficacy. (2) Refining dosage comparison. Multi-arm trials directly comparing differences in water levels are recommended to establish optimal anatomical dosages. (3) Focusing on long-term adaptation. The long-term interference of different cold therapy regions on muscle strength and hypertrophy adaptation needs to be further elucidated. This will assist practitioners in balancing acute recovery with long-term training benefits.

## 5 Conclusion

It is indicated by this systematic review and meta-analysis that post-exercise biochemical damage markers (CK) are effectively reduced and subjective pain (DOMS) is alleviated by CWI. Its clinical value as an acute recovery tool is established. However, these physiological and perceptual benefits are not synchronously translated into improvements in maximal muscle strength (MVIC). A significant inhibitory effect on explosive power (CMJ) is exhibited immediately post-exercise.

The core finding of this study is that statistical equivalence across all recovery indicators is possessed by whole-body and partial immersion. Whole-body cold exposure need not be pursued. Moreover, a clear time-dependency is presented by its positive effects, which are primarily concentrated in the 24-hour post-exercise window and tend to subside by 48–72 h. In summary, partial immersion is the optimal strategy combining efficacy and safety. It is particularly suitable for contexts aiming to accelerate next-day recovery. However, during short intervals involving continuous explosive power output, it should be used with caution or combined with active rewarming to avoid temporary impairment of neuromuscular function.

## Data Availability

The datasets presented in this study can be found in online repositories. The names of the repository/repositories and accession number(s) can be found in the article/Supplementary Material.
